# Supplementary material for: Multiomics analysis identifies VPA-induced changes in neural progenitor cells, ventricular-like regions, and cellular microenvironment in dorsal forebrain organoids
Source: Mol Psychiatry. 2026 Apr 24;31(9):4977–94. doi: 10.1038/s41380-026-03585-5 (PMC13441967; doi:10.1038/s41380-026-03585-5)
Supplement: Supplementary file 1 [file 41380_2026_3585_MOESM1_ESM.pdf]

Supplementary figures and figure legends

Figure S1

A **Experimental scheme**

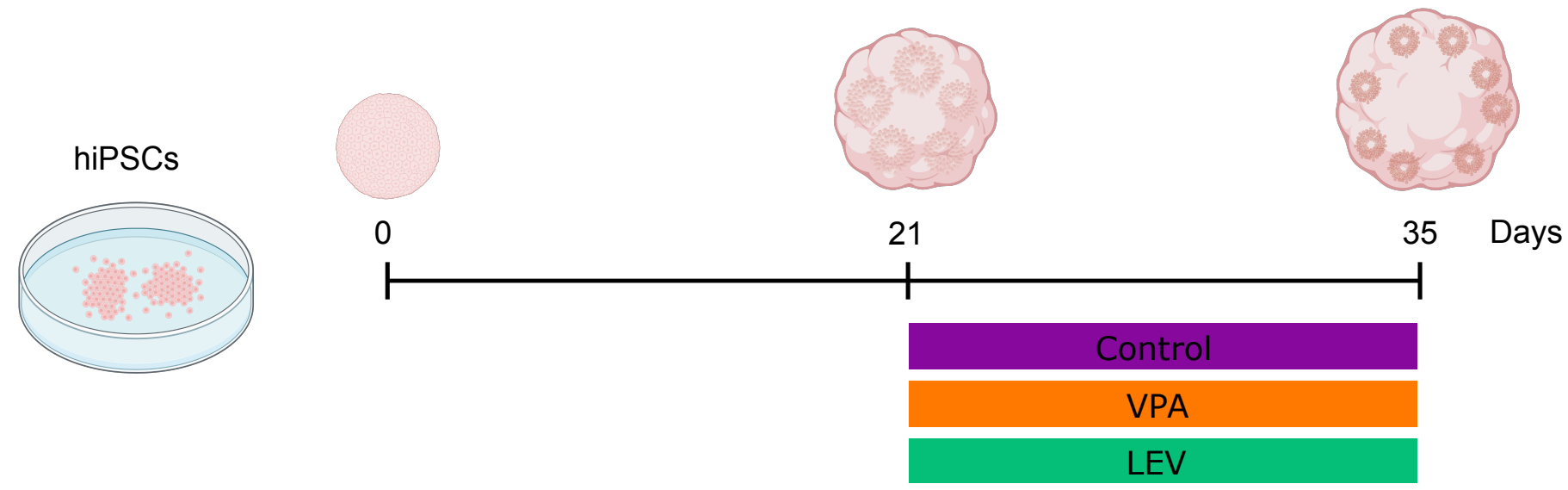

B **Bright-field images of DFOs over time**

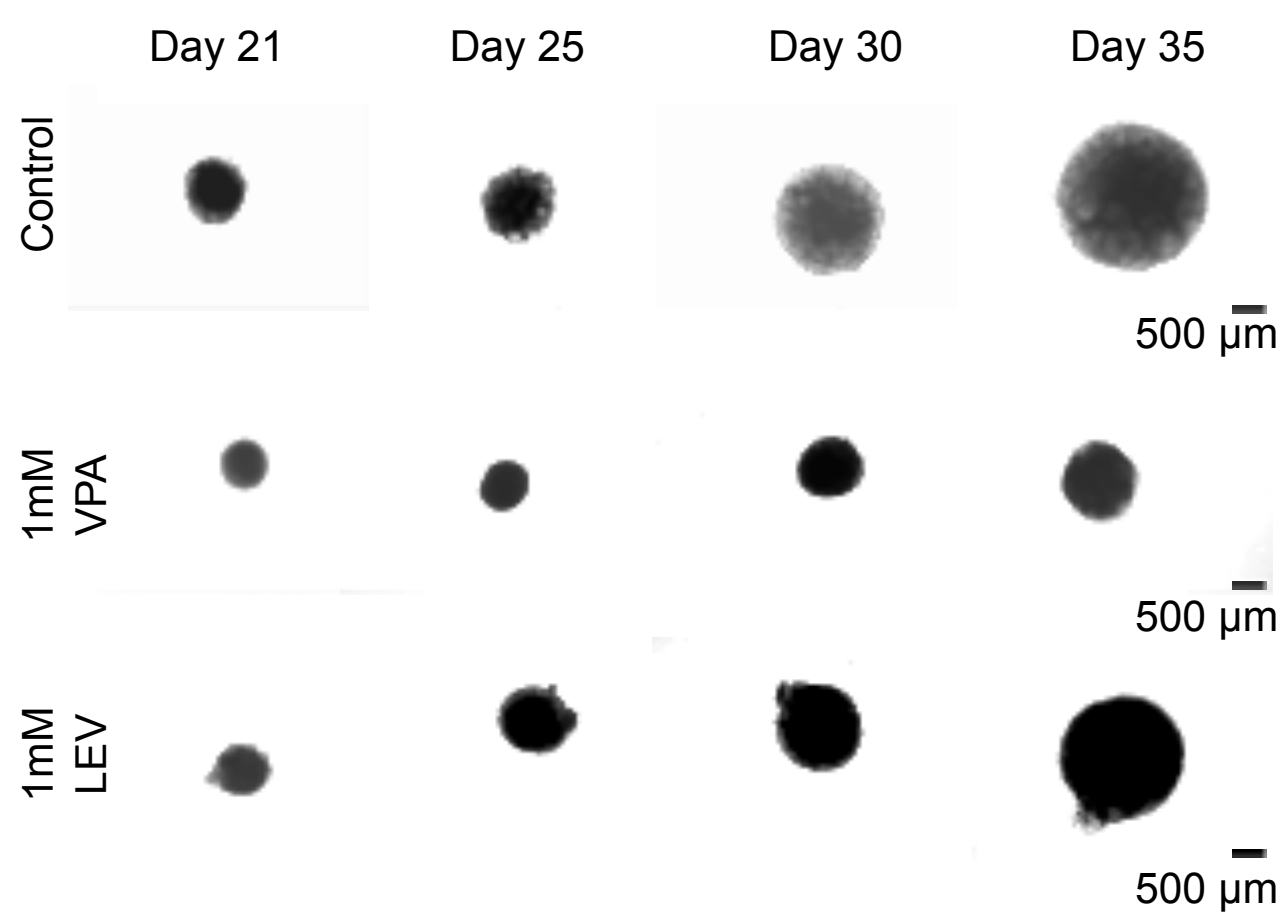

C **Size analysis of DFO treated with VPA (left) or LEV (right)**

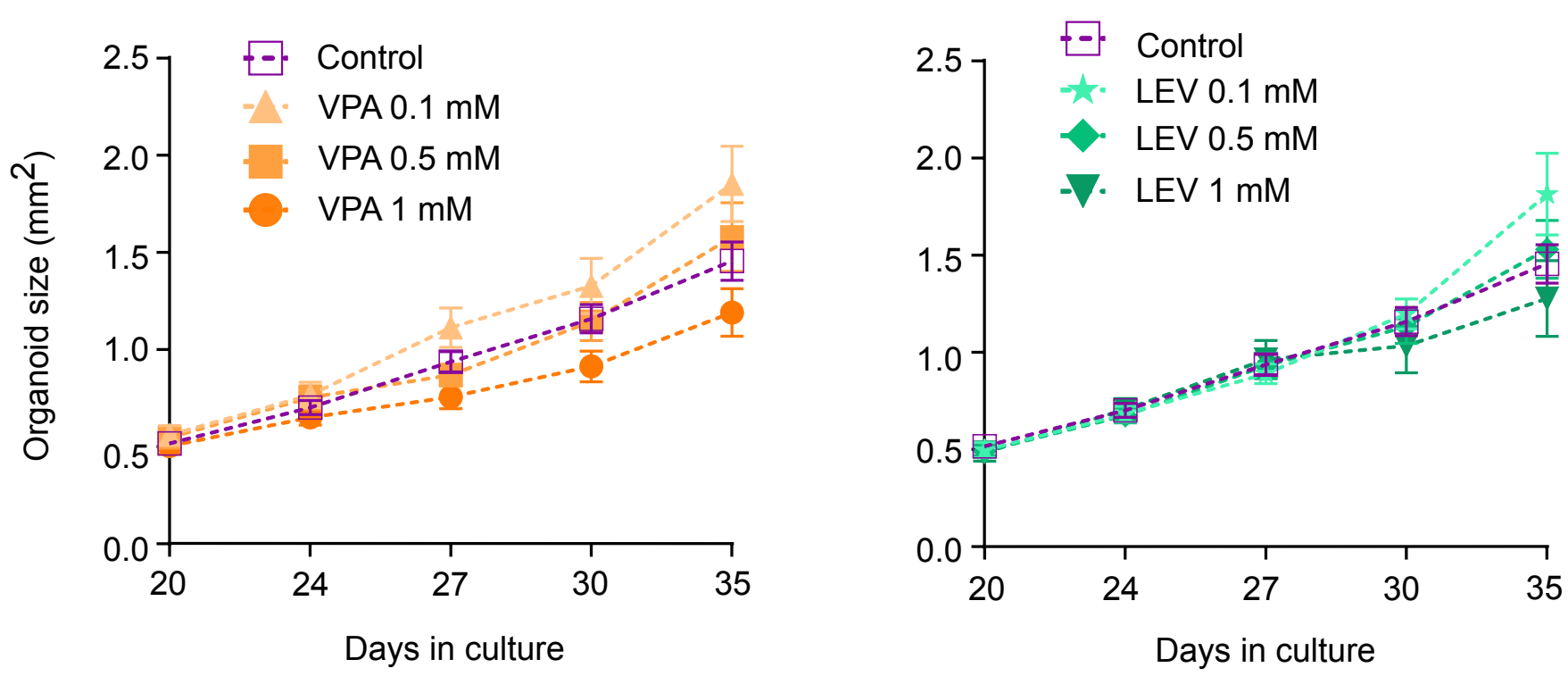

Fig. S1: Dorsal forebrain organoids to study the effects of different antiepileptic drugs (AEDs).

A. Schematic of dorsal forebrain organoid generation (1) and AED treatment from day 21-35. Ctrl, no treatment (purple); VPA, Valproate treatment (orange); and LEV, Levetiracetam treatment (green). Three individual DFO batches were generated using two different iPSC cell lines (BIONi010-C and HMGU1).
B. Representative light microscope images of organoid development during treatment period for Ctrl, 1 mM VPA and 1mM LEV.
C. Quantification of organoid size throughout treatment period with VPA compared to Ctrl. Data points represent the mean of all data in each condition, and the error bars represent the standard error of mean (SEM). For all concentrations of VPA and LEV at D20, D24, D30 and D35, n=14 (except for 1 mM VPA and 0.5 mM VPA at D35 n=13; 0.5 mM LEV at D20 n=13) and at D27 n=10; for Ctrl at D20 and D35 n=29, at D24 n=30, D27 n= 22 and at D30 n=28. One-way ANOVA test with Bonferroni correction for multiple comparisons was used for significance testing between VPA and Ctrl per time point and no significance was found for any treatment to Ctrl comparisons.

Figure S2

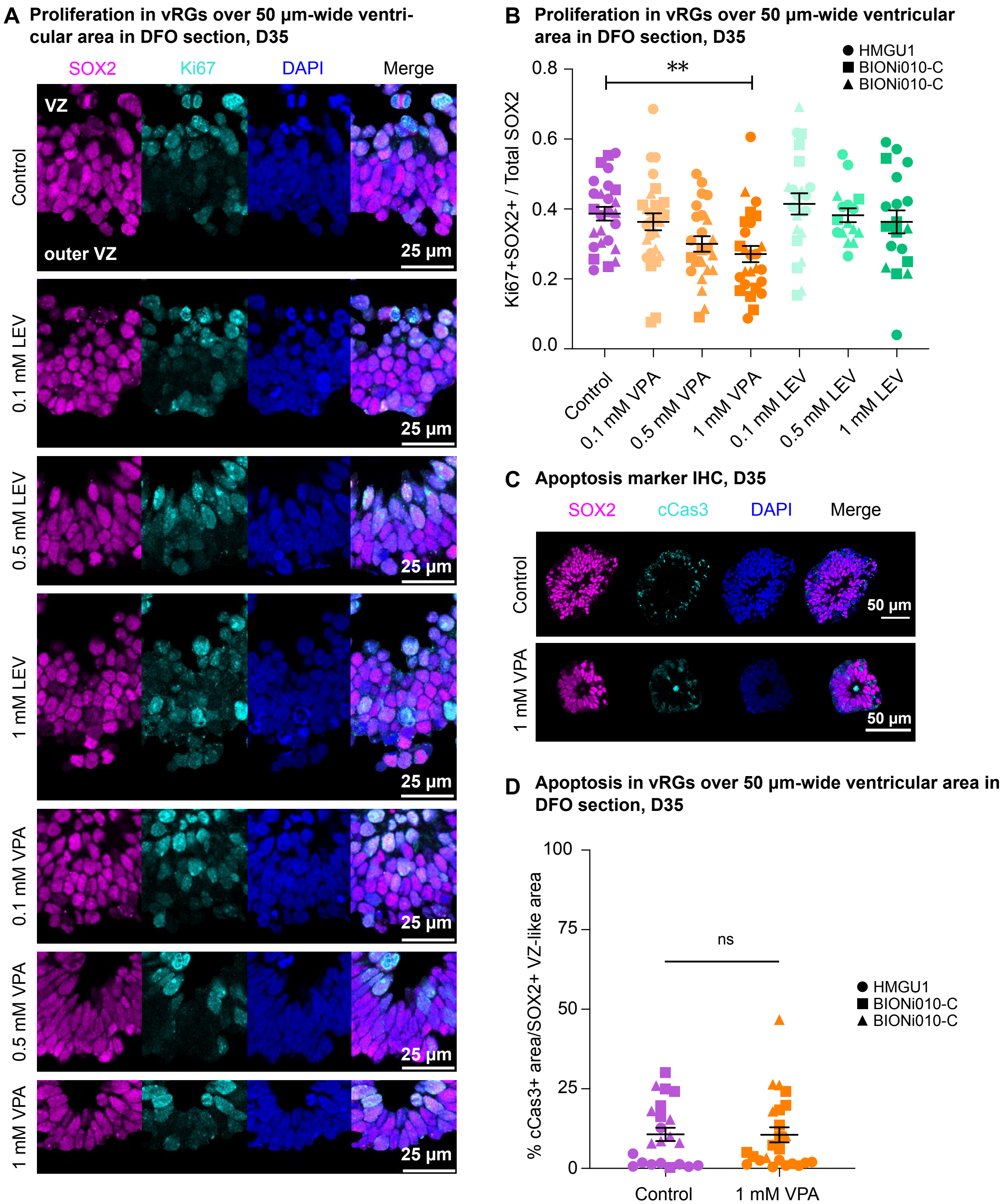

A Proliferation in vRGs over 50 µm-wide ventricular area in DFO section, D35
SOX2
Ki67
DAPI
Merge
Control
VZ
outer VZ
0.1 mM LEV
0.5 mM LEV
1 mM LEV
0.1 mM VPA
0.5 mM VPA
1 mM VPA
25 µm
B Proliferation in vRGs over 50 µm-wide ventricular area in DFO section, D35
● HMGU1
■ BIONi010-C
▲ BIONi010-C
Ki67+SOX2+ / Total SOX2
0.8
0.6
0.4
0.2
0.0
**
Control
0.1 mM VPA
0.5 mM VPA
1 mM VPA
0.1 mM LEV
0.5 mM LEV
1 mM LEV
C Apoptosis marker IHC, D35
SOX2
cCas3
DAPI
Merge
Control
1 mM VPA
50 µm
D Apoptosis in vRGs over 50 µm-wide ventricular area in DFO section, D35
% cCas3+ area/SOX2+ VZ-like area
100
75
50
25
0
ns
● HMGU1
■ BIONi010-C
▲ BIONi010-C
Control
1 mM VPA

Fig. S2: Changes in proliferation and apoptosis upon VPA treatment at D35.
A. 50 μm-wide cropped confocal image of a representative VZ-like region from different treatment conditions. RG cells (magenta) and proliferative cells (cyan).
B. Quantification of Ki67+SOX2+ over total SOX2+ cells in this region for each treatment condition (Ctrl, n=26; 0.1 mM VPA, n=28; 0.5 mM VPA, n=24; 1 mM VPA, n=26; 0.1 mM LEV, n=22; 0.5 mM LEV, n=16; 1 mM LEV, n=19). The shape of the individual data points represents different batches, therefore the cell line of the DFO differentiation. One-way ANOVA statistical test was performed with Bonferroni correction for multiple testing, only comparison between Ctrl vs. 1 mM VPA was significant (p=0.0039).
C. Representative image of DFO section for SOX2+ RG cells (magenta) and cCas3+ apoptotic cells (cyan).
D. Quantification of apoptosis in vRG cells in 50 μm-wide ventricular area in DFO slice at D35. Nonparametric Mann-Whitney test was performed for statistical analysis and showed no significance between Ctrl (n=22) and 1 mM VPA (n=24) groups. Shapiro-Wilk test was used to assess normality.

Figure S3

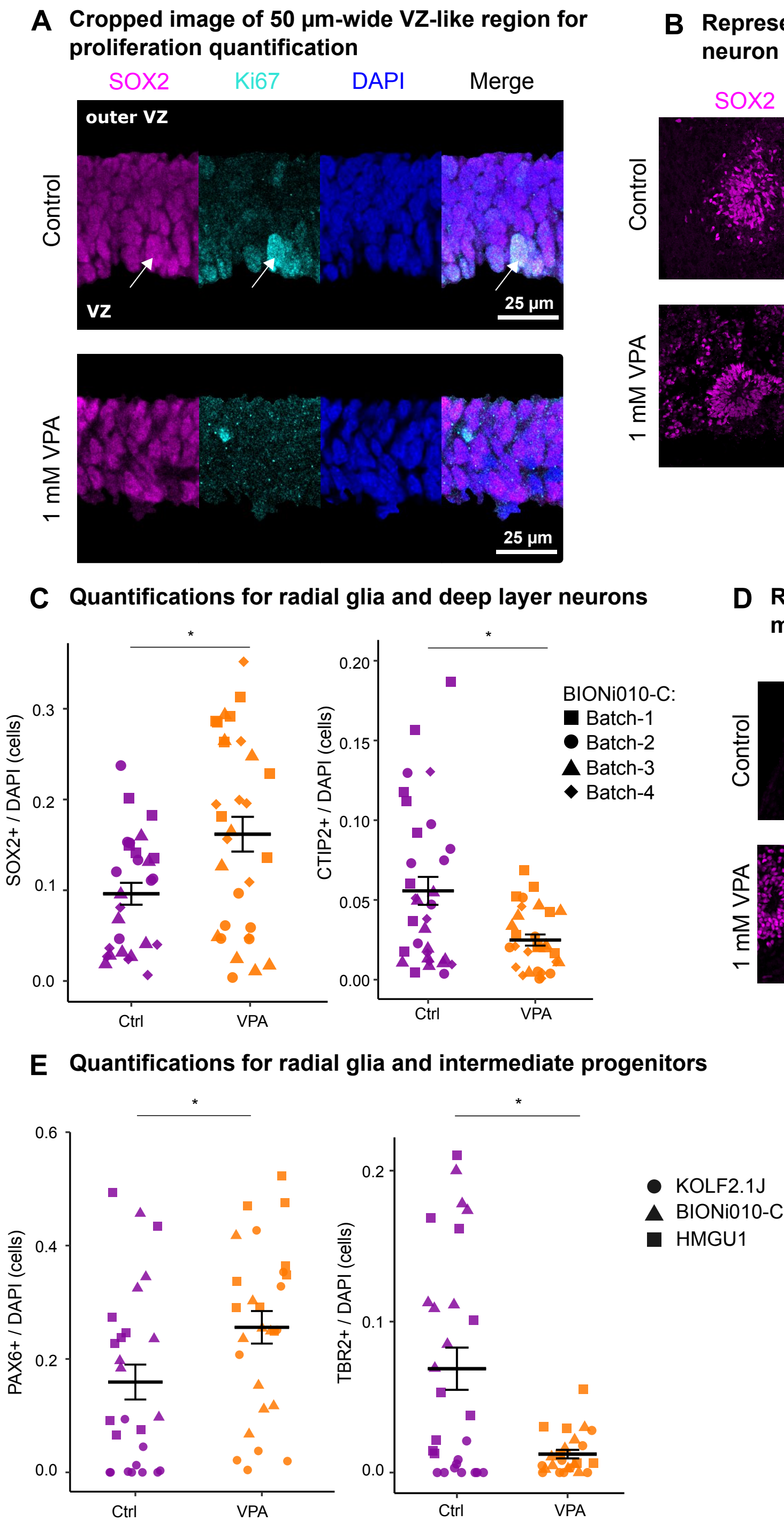

A Cropped image of 50 µm-wide VZ-like region for proliferation quantification
SOX2
Ki67
DAPI
Merge
outer VZ
VZ
Control
1 mM VPA
25 µm
B Representative image for radial glia and deep layer neuron quantification
SOX2
CTIP2
DAPI
Merge
Control
1 mM VPA
50 µm
C Quantifications for radial glia and deep layer neurons
SOX2+ / DAPI (cells)
CTIP2+ / DAPI (cells)
Ctrl
VPA
*
BIONi010-C:
Batch-1
Batch-2
Batch-3
Batch-4
D Representative image for radial glia and intermediate progenitor
PAX6
TBR2
DAPI
Merge
Control
1 mM VPA
50 µm
E Quantifications for radial glia and intermediate progenitors
PAX6+ / DAPI (cells)
TBR2+ / DAPI (cells)
Ctrl
VPA
*
KOLF2.1J
BIONi010-C
HMGU1

Fig. S3: Dorsal forebrain organoids to study the effects of long-term exposure to VPA.

A. Representative 50 µm-wide cropped images of VZ-like region for the quantification of Ki67+SOX2+/Total SOX2+ cells in Ctrl and VPA-treated DFOs. SOX2+ RG cells (magenta) and Ki67+ proliferative cells (cyan). An example of double positive Ki67+SOX2+ cell is shown using a white arrow.

B. Representative images of VZ-like region for the quantification of SOX2+/DAPI+ cells and CTIP2+/DAPI+ cells in Ctrl and VPA-treated DFOs. SOX2+ RG cells (magenta) and CTIP2+ deep-layer neurons (cyan).

C. Quantification of SOX2+/DAPI+ cells (left, Ctrl (n = 28) and VPA-treated (n = 31)) and CTIP2+/DAPI+ cells (right, Ctrl (n = 31) and VPA-treated (n = 31)) DFOs. The colors represent the experimental group (Ctrl, purple; VPA, orange) and shape of the data points represent the DFO differentiation batch. Outliers were removed based on interquartile range method. Nonparametric Mann-Whitney test was performed for statistical analysis. The comparison between Ctrl vs. VPA was significant for SOX2+/DAPI+ cells ($p = 0.0155$) and CTIP2+/DAPI+ cells ($p = 0.0176$).

D. Representative images of VZ-like region for the quantification of PAX6+/DAPI+ cells and TBR2+/DAPI+ cells in Ctrl and VPA-treated DFOs. PAX6+ RG cells (magenta) and TBR2+ intermediate progenitor cells (cyan).

E. Quantification of PAX6+/DAPI+ cells (left, Ctrl (n = 26) and VPA-treated (n = 27)) and TBR2+/DAPI+ cells (right, Ctrl (n = 27) and VPA-treated (n = 24)) DFOs. The colors represent the experimental group (Ctrl, purple; VPA, orange) and shape of the data points represent the DFO differentiation batch. Outliers were removed based on interquartile range method. Nonparametric Mann-Whitney test was performed for statistical analysis. The comparison between Ctrl vs VPA was significant for PAX6+/DAPI+ cells ($p = 0.0106$), and for TBR2+/DAPI+ cells ($p = 0.0173$).

Figure S4

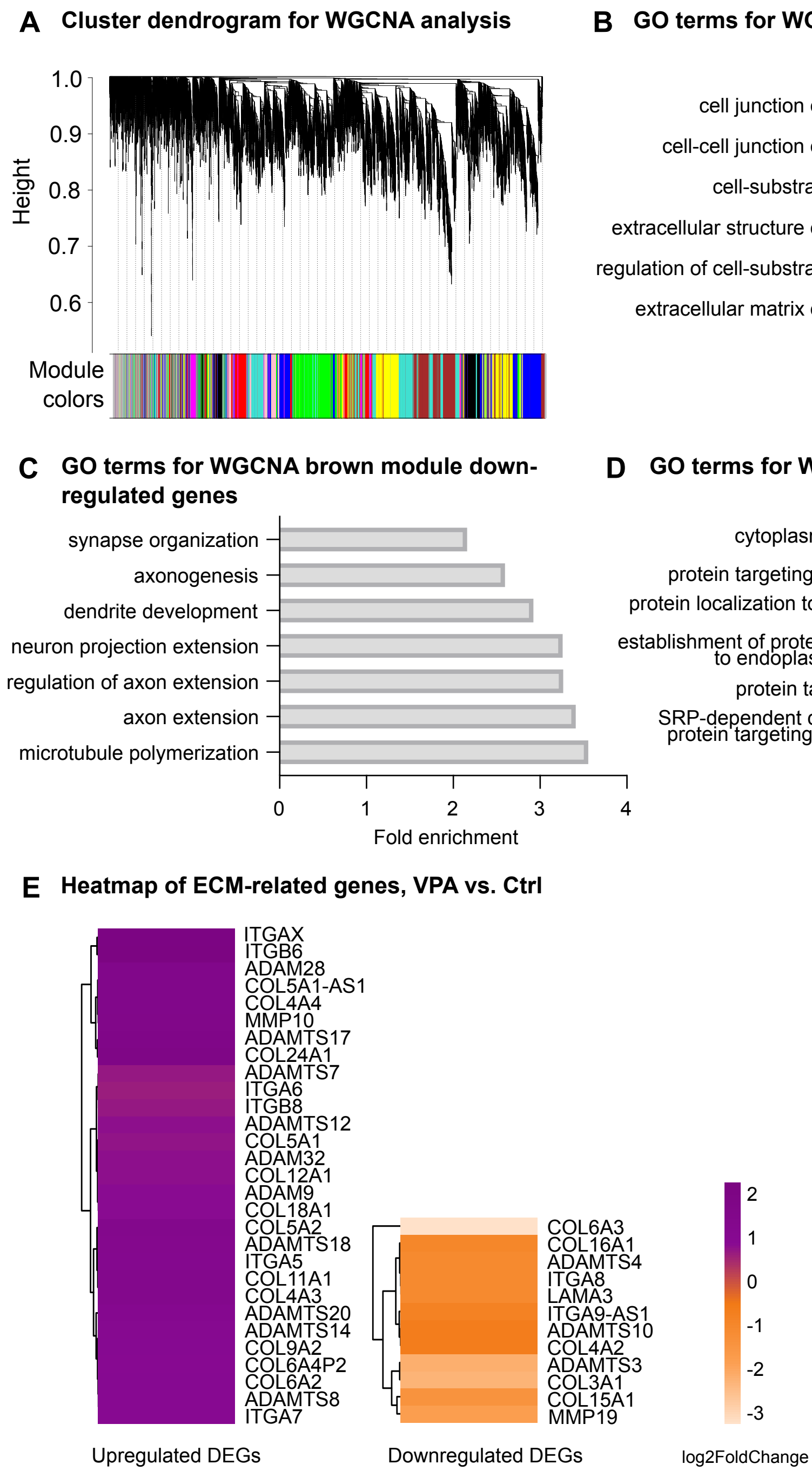
A Cluster dendrogram for WGCNA analysis
1.0
0.9
0.8
0.7
0.6
Height
Module colors
B GO terms for WGCNA yellow module upregulated genes
cell junction organization
cell-cell junction organization
cell-substrate adhesion
extracellular structure organization
regulation of cell-substrate adhesion
extracellular matrix organization
0 1 2 3
Fold enrichment
C GO terms for WGCNA brown module down-regulated genes
synapse organization
axonogenesis
dendrite development
neuron projection extension
regulation of axon extension
axon extension
microtubule polymerization
0 1 2 3 4
Fold enrichment
D GO terms for WGCNA black module downregulated genes
cytoplasmic translation
protein targeting to membrane
protein localization to endoplasmic reticulum
establishment of protein localization to endoplasmic reticulum
protein targeting to ER
SRP-dependent cotranslational protein targeting to membrane
0 5 10 15 20 25
Fold enrichment
E Heatmap of ECM-related genes, VPA vs. Ctrl
ITGAX
ITGB6
ADAM28
COL5A1-AS1
COL4A4
MMP10
ADAMTS17
COL24A1
ADAMTS7
ITGA6
ITGB8
ADAMTS12
COL5A1
ADAM32
COL12A1
ADAM9
COL18A1
COL5A2
ADAMTS18
ITGA5
COL11A1
COL4A3
ADAMTS20
ADAMTS14
COL9A2
COL6A4P2
COL6A2
ADAMTS8
ITGA7
COL6A3
COL16A1
ADAMTS4
ITGA8
LAMA3
ITGA9-AS1
ADAMTS10
COL4A2
ADAMTS3
COL3A1
COL15A1
MMP19
2
1
0
-1
-2
-3
Upregulated DEGs
Downregulated DEGs
log2FoldChange

Fig. S4: WGCNA network analysis with RNA-sequencing data.
A. Dendrogram for the WGCNA to identify the cluster of genes with similar expression patterns.
B. Selected GO terms for biological processes associated with upregulated genes in the WGCNA yellow module.
C. Selected GO terms for biological processes associated with downregulated genes in the WGCNA brown module.
D. Selected GO terms for biological processes associated with downregulated genes in the WGCNA black module.
E. Heatmap of upregulated (left) and downregulated (right) collagen, laminin, integrin and metalloprotease genes in VPA vs. Ctrl DFOs.

Figure S5

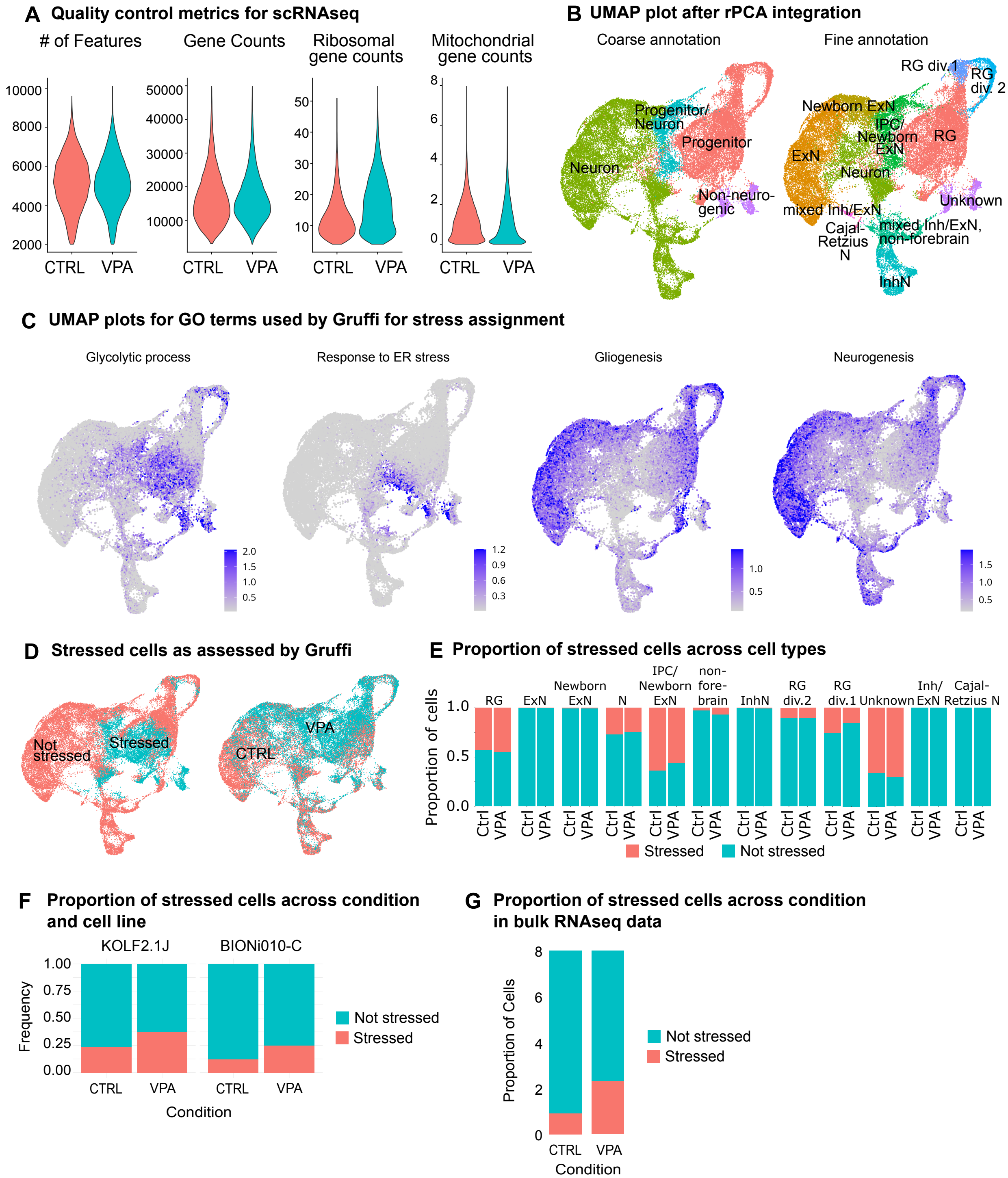

A Quality control metrics for scRNAseq
# of Features
Gene Counts
Ribosomal gene counts
Mitochondrial gene counts
CTRL
VPA
B UMAP plot after rPCA integration
Coarse annotation
Fine annotation
Progenitor/ Neuron
Progenitor
Neuron
Non-neuro-genic
RG div.1
RG div. 2
Newborn ExN
IPC/ Newborn ExN
RG
ExN
Unknown
mixed Inh/ExN
Cajal-Retzius N
mixed Inh/ExN, non-forebrain
InhN
C UMAP plots for GO terms used by Gruffi for stress assignment
Glycolytic process
Response to ER stress
Gliogenesis
Neurogenesis
D Stressed cells as assessed by Gruffi
Not stressed
Stressed
CTRL
VPA
E Proportion of stressed cells across cell types
Proportion of cells
RG
ExN
Newborn ExN
N
IPC/ Newborn ExN
non-fore-brain
InhN
RG div.2
RG div.1
Unknown
Inh/ExN
Cajal-Retzius N
Ctrl
VPA
Stressed
Not stressed
F Proportion of stressed cells across condition and cell line
KOLF2.1J
BIONi010-C
Frequency
Condition
Not stressed
Stressed
G Proportion of stressed cells across condition in bulk RNAseq data
Proportion of Cells
Condition
Not stressed
Stressed

Fig. S5: Quality control measurements for scRNA-seq.
A. Violin plot of QC metrics, including number of features, number of gene counts, ribosomal gene counts and mitochondrial gene counts after filtering.
B. UMAP plot with crude cell type annotations after reciprocal PCA integration of the data.
C. UMAP plots of GO terms (positive markers of stress: glycolytic process, response to ER; negative markers of stress: gliogenesis and neurogenesis) used by Gruffi for stress assignment.
D. Stressed cell assessment by Gruffi. Distribution of the stressed (cyan) and not stressed (red) cells on UMAP (top left), distribution of conditions on UMAP (top right).
E. The proportion of stressed cells within each cell type. Stressed (cyan) and not stressed (red) cells.
F. The proportion of stressed cells across experimental condition and cell line: KOLF2.1J (left) and BIONi010-C (right). Stressed (cyan) and not stressed (red) cells.
G. Assessment of stressed cells across experimental condition in bulk RNAseq data. Stressed (cyan) and not stressed (red) cells.

Figure S6

A **Regional identity using VoxHunt - E18 mouse tissue**

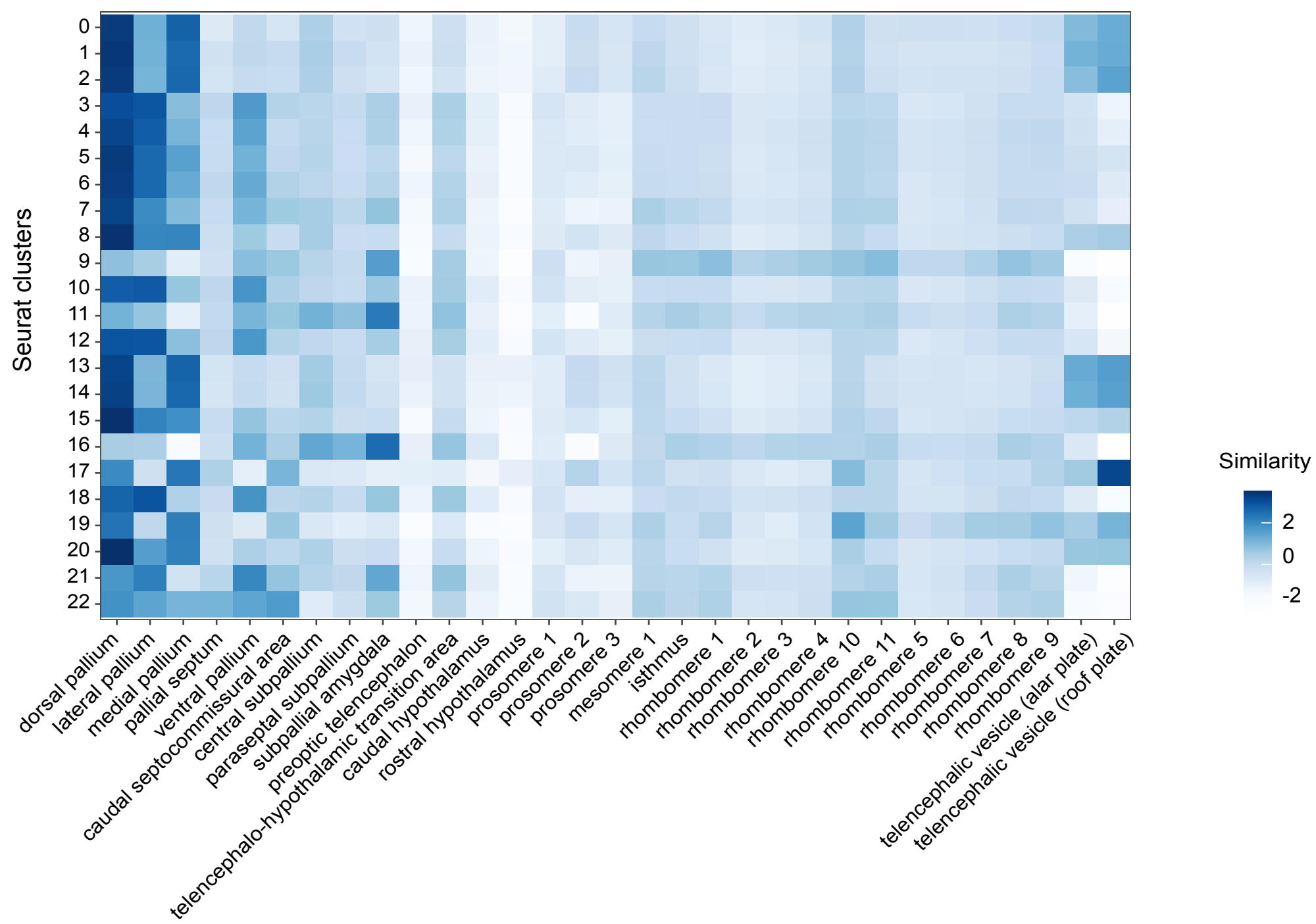

B **Regional identity using ArchMap - human fetal brain tissue**

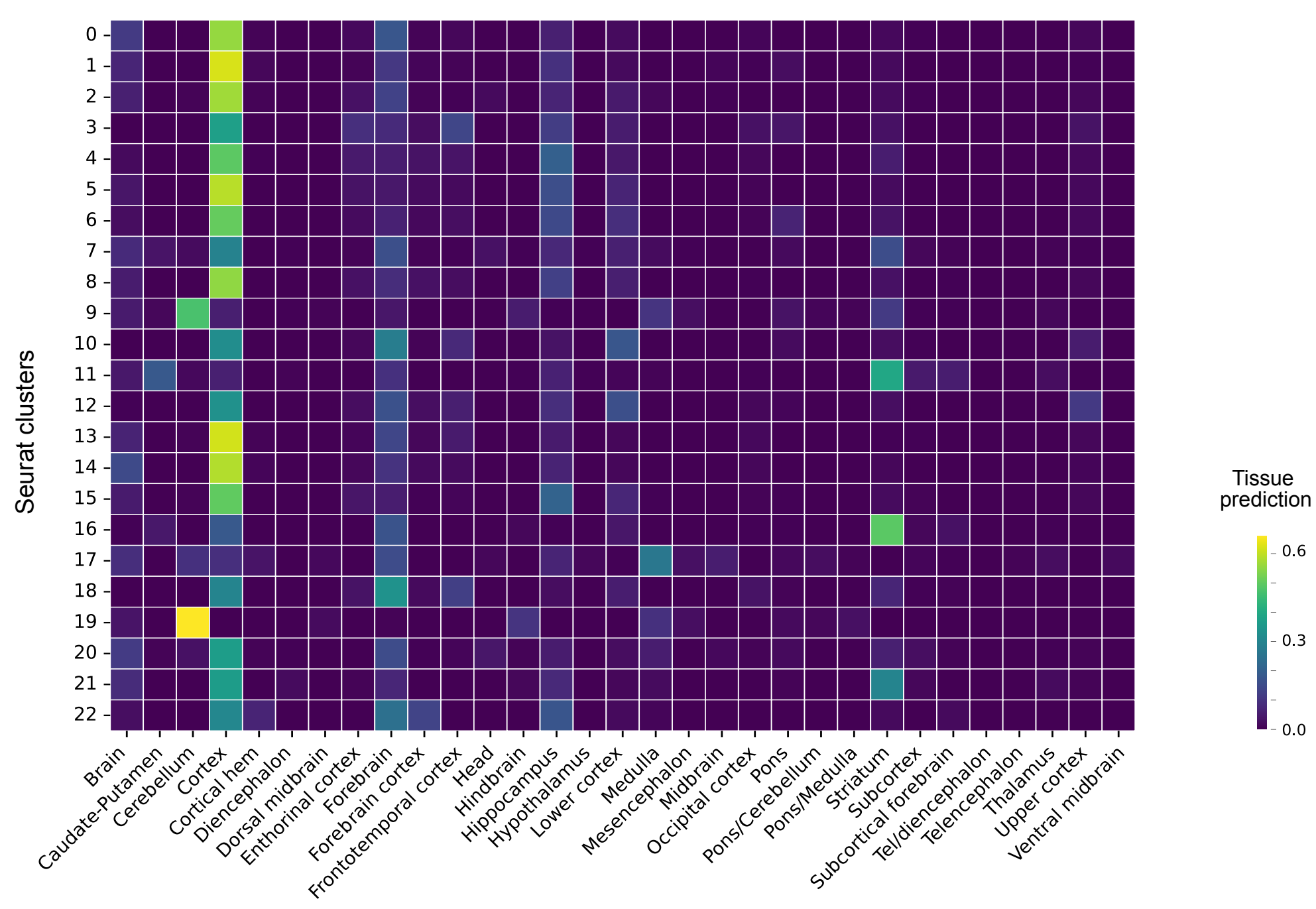

Fig. S6: Regional identity of DFOs at D50.
A. Regional identity of individual clusters using VoxHunt to identify non-forebrain clusters. Mouse E18 dataset has been used.
B. Regional identity of individual clusters using ArchMap to identify non-forebrain clusters. Human fetal brain tissue dataset has been used.

Figure S7

A UMAP plots for GO terms used by Gruffi after removal of stressed and non-forebrain cells

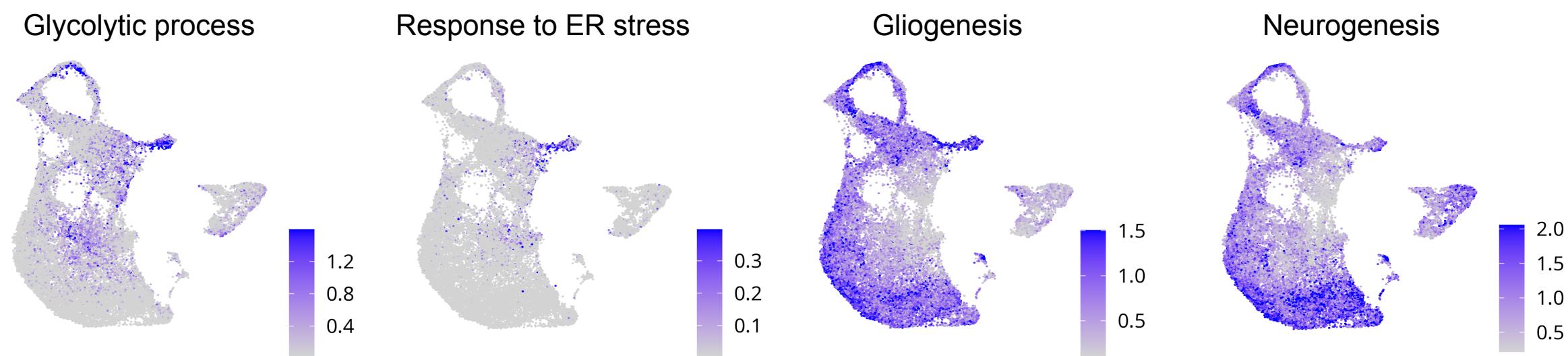

B Technical features after removal of stressed and non-forebrain cells

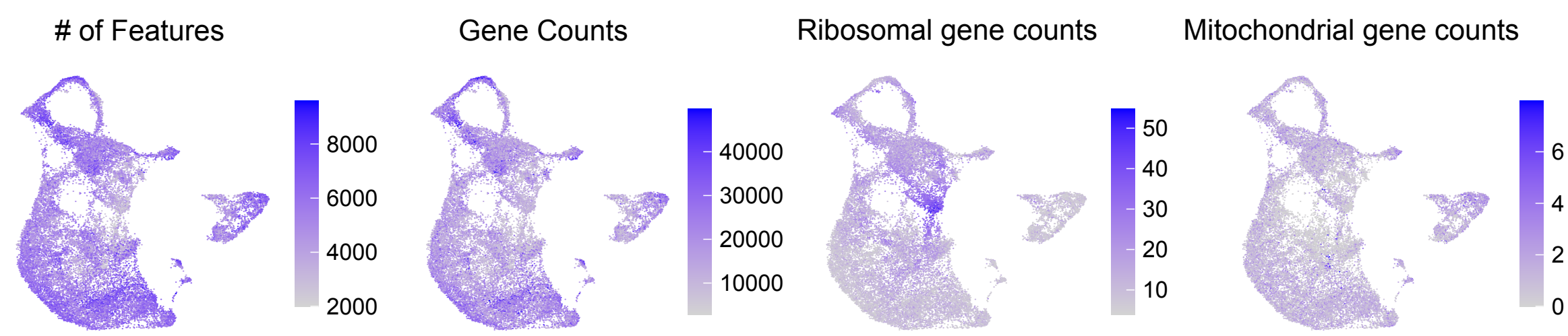

C UMAP plot after removal of stressed and non-forebrain cells

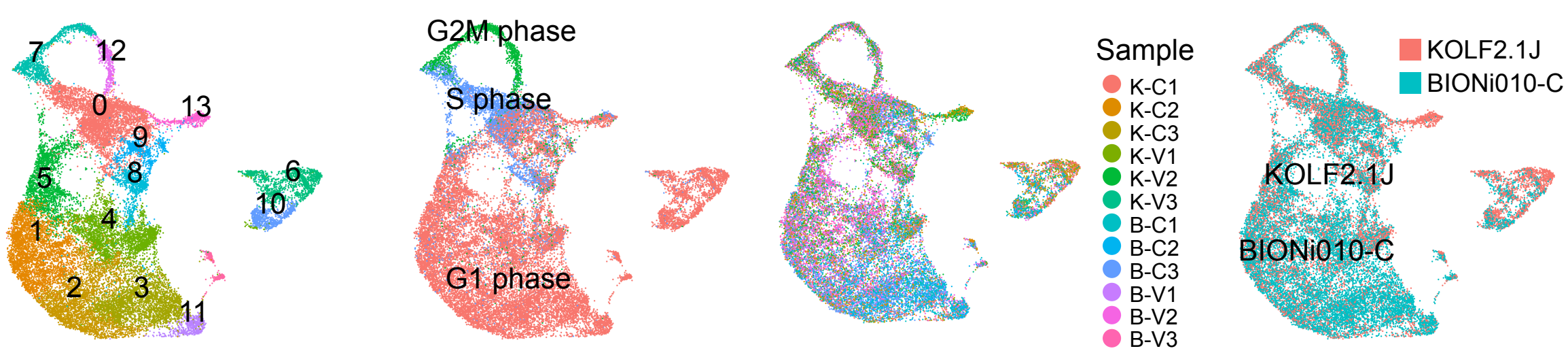

Fig. S7: Features of the scRNAseq data after removal of stressed and non-forebrain cells.

A. UMAP plots of GO terms (positive markers of stress: glycolytic process, response to ER; negative markers of stress: gliogenesis and neurogenesis) used by Gruffi after removal of stressed and non-forebrain cells.

B. UMAP plots showing technical features, including number of RNA features (leftmost), RNA count (middle-left), ribosomal RNA counts (middle-right), and mitochondrial RNA counts (rightmost) after removal of stressed cells.

C. UMAP plot of normalized cells after integration by sample, with annotations for new cluster numbers (leftmost), cell cycle phase (middle-left), distribution of cells from different samples (middle-right), and distribution of cells from different cell lines (BIONi010-C and KOLF2.1J) (rightmost), following the removal of off-target and stressed cells.

Figure S8

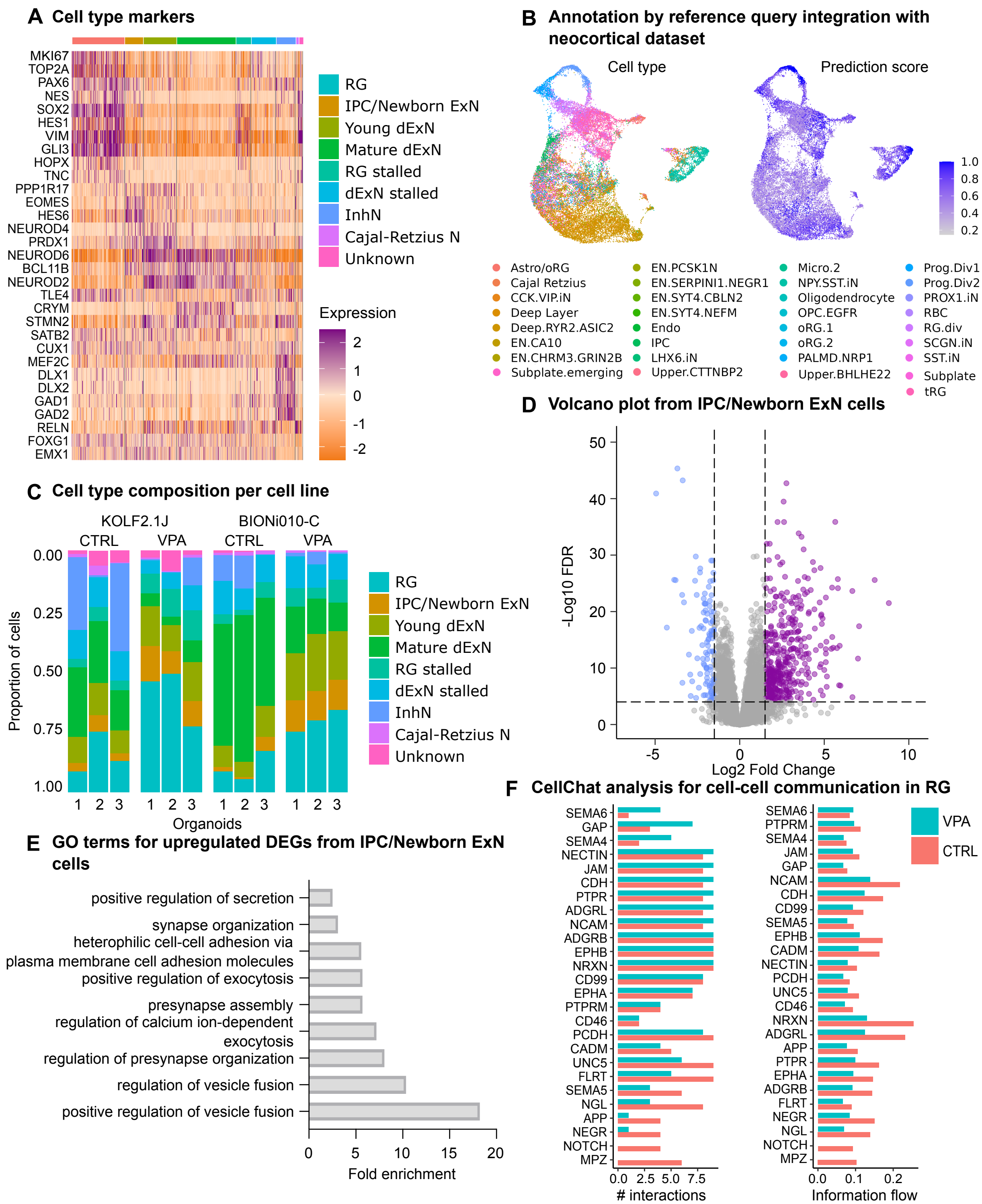

A Cell type markers
MKI67
TOP2A
PAX6
NES
SOX2
HES1
VIM
GLI3
HOPX
TNC
PPP1R17
EOMES
HES6
NEUROD4
PRDX1
NEUROD6
BCL11B
NEUROD2
TLE4
CRYM
STMN2
SATB2
CUX1
MEF2C
DLX1
DLX2
GAD1
GAD2
RELN
FOXG1
EMX1
RG
IPC/Newborn ExN
Young dExN
Mature dExN
RG stalled
dExN stalled
InhN
Cajal-Retzius N
Unknown
Expression
B Annotation by reference query integration with neocortical dataset
Cell type
Prediction score
Astro/oRG
Cajal Retzius
CCK.VIP.iN
Deep Layer
Deep.RYR2.ASIC2
EN.CA10
EN.CHRM3.GRIN2B
Subplate.emerging
EN.PCSK1N
EN.SERPINI1.NEGR1
EN.SYT4.CBLN2
EN.SYT4.NEFM
Endo
IPC
LHX6.iN
Upper.CTTNBP2
Micro.2
NPY.SST.iN
Oligodendrocyte
OPC.EGFR
oRG.1
oRG.2
PALMD.NRP1
Upper.BHLHE22
Prog.Div1
Prog.Div2
PROX1.iN
RBC
RG.div
SCGN.iN
SST.iN
Subplate
tRG
C Cell type composition per cell line
KOLF2.1J
BIONi010-C
CTRL
VPA
Proportion of cells
Organoids
D Volcano plot from IPC/Newborn ExN cells
-Log10 FDR
Log2 Fold Change
E GO terms for upregulated DEGs from IPC/Newborn ExN cells
positive regulation of secretion
synapse organization
heterophilic cell-cell adhesion via plasma membrane cell adhesion molecules
positive regulation of exocytosis
presynapse assembly
regulation of calcium ion-dependent exocytosis
regulation of presynapse organization
regulation of vesicle fusion
positive regulation of vesicle fusion
Fold enrichment
F CellChat analysis for cell-cell communication in RG
VPA
CTRL
interactions
Information flow

Fig. S8: Cell type annotation and cell-type specific analysis for scRNA-seq.
A. Heatmap of manually selected cell type markers used for cell type annotation.
B. UMAP plot showing cell type annotations based on reference-query integration with a neocortical development dataset (left) and the respective confidence scores (right). As reference dataset, integrated fetal transcriptomic dataset was used (2).
C. Stacked bar plot with the proportion of cell types per sample. All samples from different cell lines and conditions were plotted individually.
D. Volcano plot of DEGs in IPC/Newborn ExN cells comparing transcriptome of VPA-treated DFOs to Ctrl (significance threshold: absolute log2 Fold change > 1.5 and FDR < 0.0001). Each datapoint represents a gene; upregulated DEGs are colored in purple, downregulated DEGs are in blue and nonsignificant genes are in grey.
E. Bar plot showing selected biological processes derived from GO analysis using significantly upregulated DEGs between IPC/Newborn ExN cells from VPA and Ctrl DFOs.
F. CellChat analysis of cell-cell communication comparing VPA and Ctrl transcriptomes. Bar plot of interaction strength (left) and relative information flow (right). VPA in cyan, Ctrl in red.

Figure S9

A **Regulons in VPA vs. Ctrl in RGs identified by SCENIC analysis**

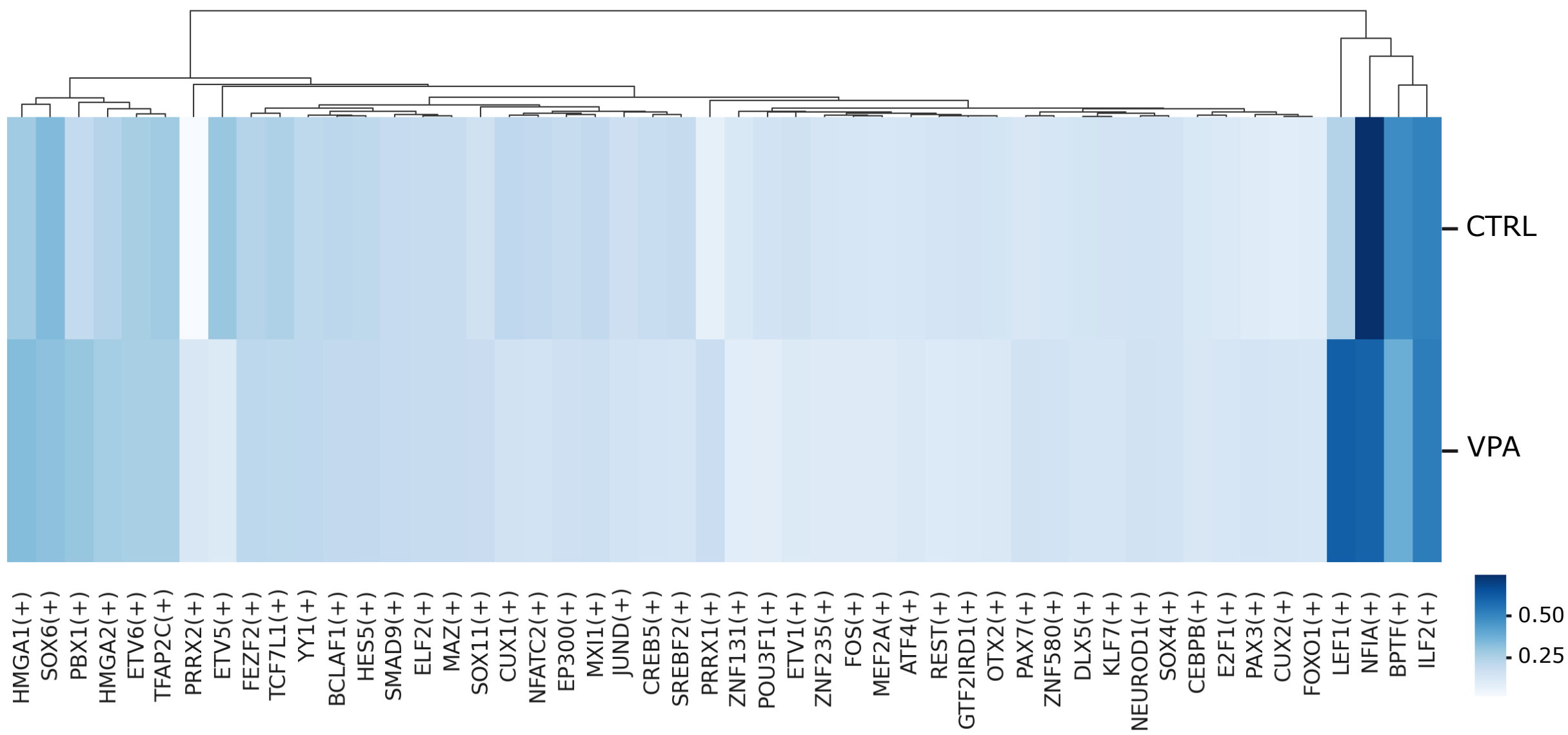

B **Relevance of VPA-dependent gene expression changes to neurodevelopmental risk genes curated by Mato-Blanco et. al (2025)**

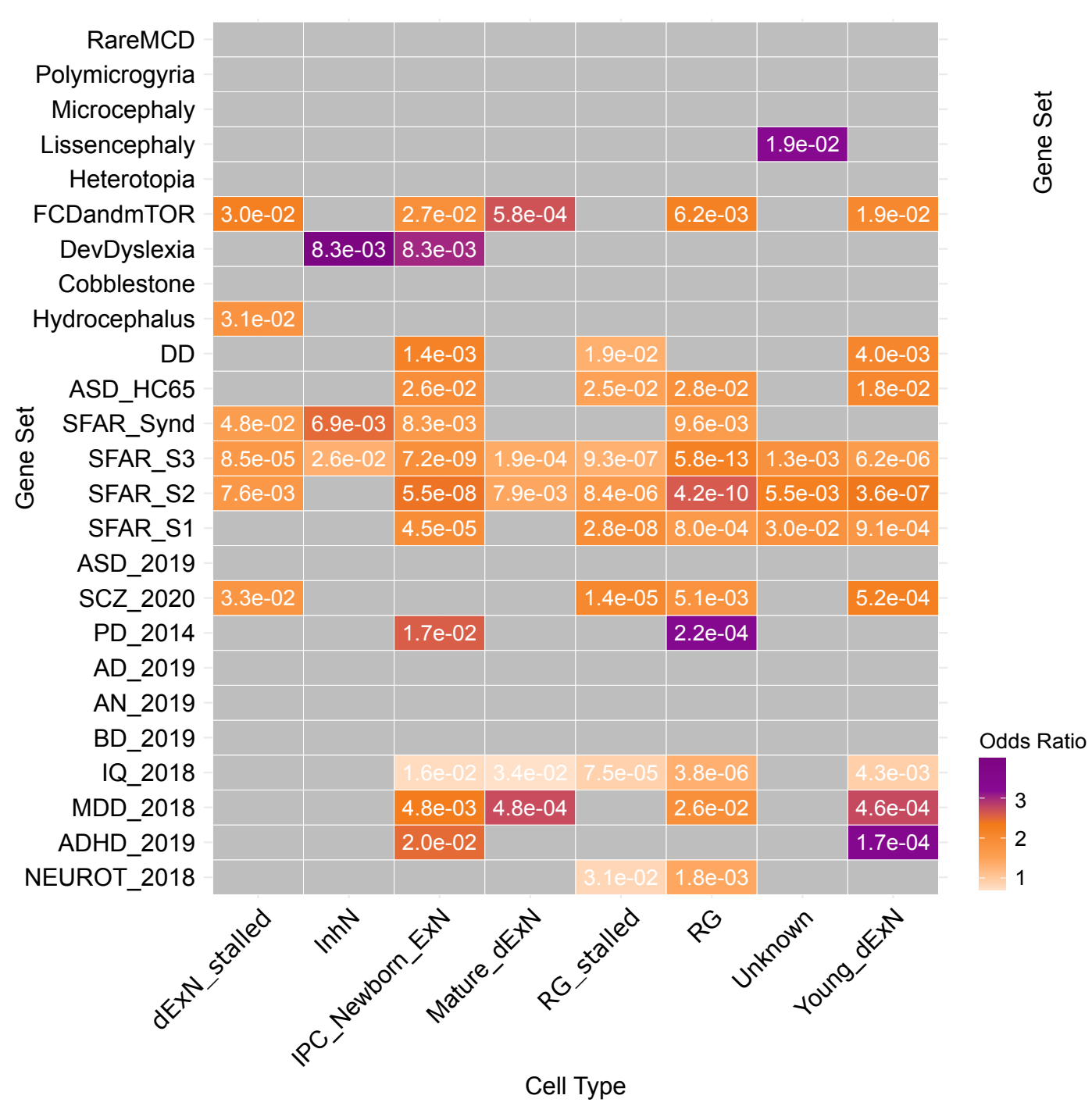

C **Relevance of VPA-dependent gene expression changes to ASD**

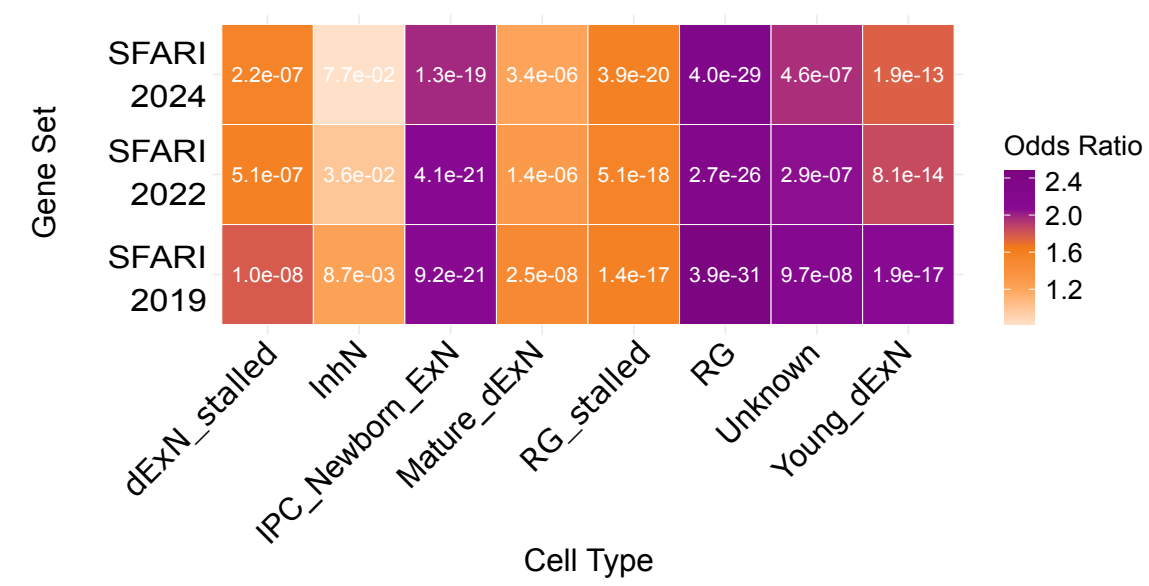

Fig. S9: Transcriptomic analysis of VPA-treated and Ctrl DFOs.
A. Heatmap showing the mean AUC scores of the top 50 most active regulons in RG cells as assessed with SCENIC, and across experimental conditions, VPA and Ctrl.
B. Heatmap displaying the enrichment of neurodevelopmental disorders-related gene sets in VPA-treated cell types. Gene sets are categorized based on a published gene list (3). The grey fill color has been used for non-significant intersections between a gene set and cell types.
C. Heatmap displaying the enrichment of ASD-related gene sets in VPA-treated cell types. Gene sets are categorized as SFARI 2019, 2022, and 2024.

For **B** and **C**, the enrichment is calculated based on the log2 odds ratio from Fisher's exact test, with p-values annotated on each tile. Significant enrichment is shown across multiple cell types and gene sets, filtered by FDR < 0.0001 and log2FC > 1.5. Colors represent the magnitude of enrichment (log2 odds ratio).

Figure S10

A **PCA plot for organoid proteome samples**

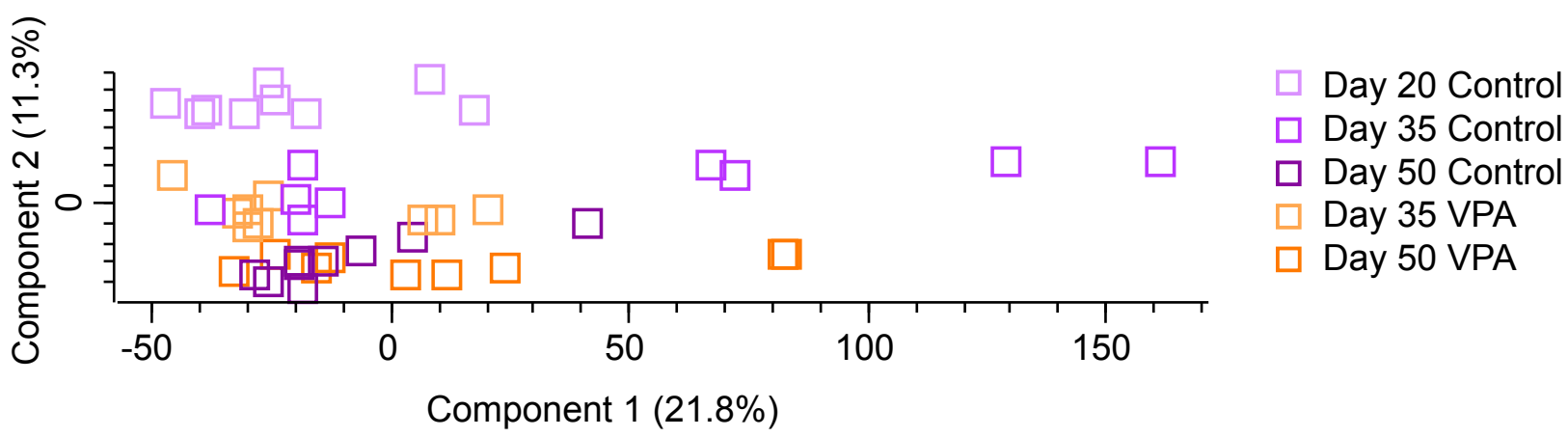

B **Volcano plot for VPA vs. Ctrl proteome at D35**

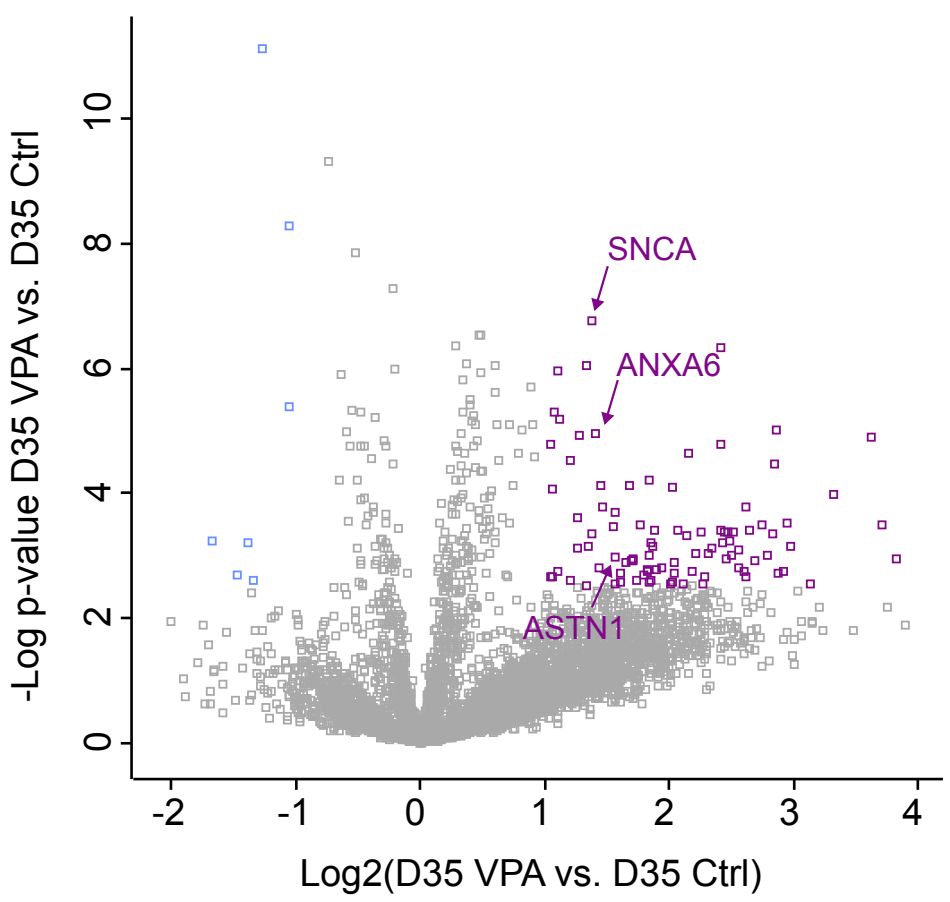

C **Volcano plot for VPA vs. Ctrl proteome at D50**

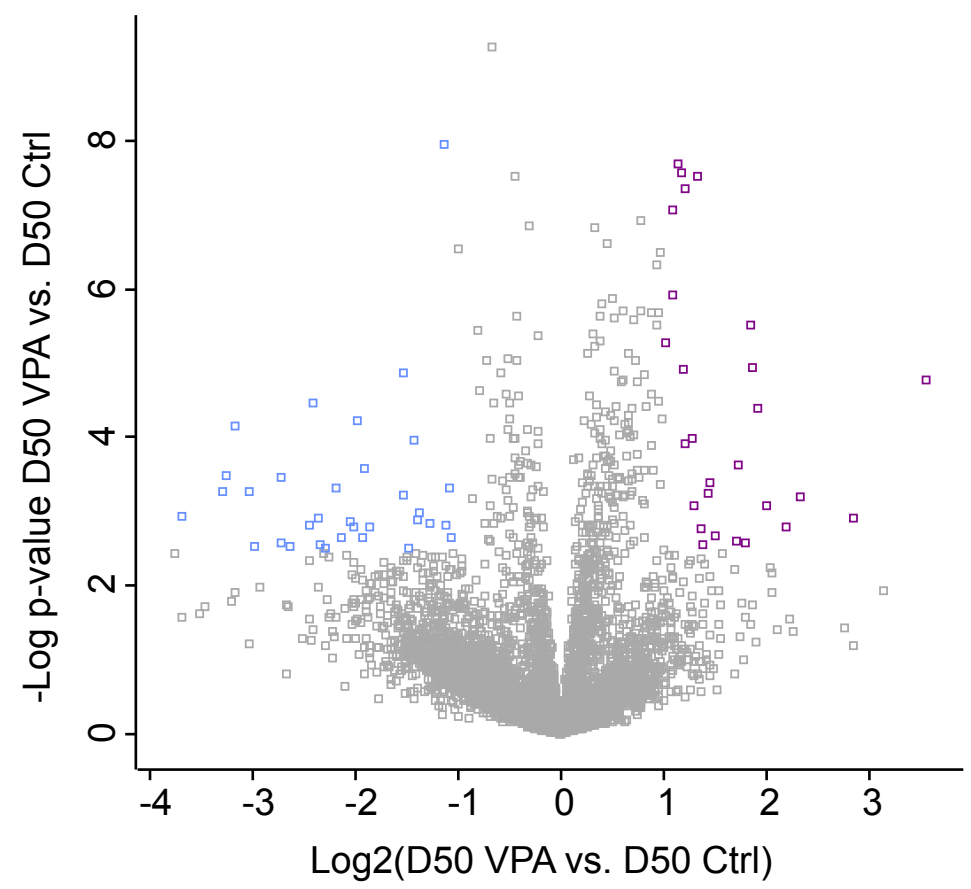

D **Number of differentially abundand proteins VPA vs. Ctrl proteome**

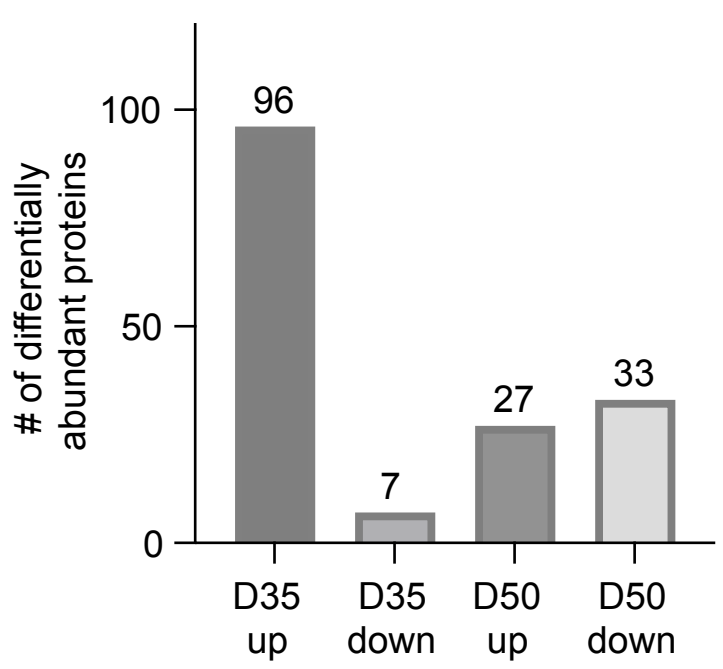

Fig. S10: Proteome analysis of DFOs treated with VPA compared to Ctrl.
A. PCA plot of all VPA-treated (orange) and Ctrl (purple) proteome samples from D20, D35 and D50. Each datapoint represents one sample, n=9 per time point. As shown in Fig. 5A, DFOs were treated with 1 mM VPA from D21 until D50. BIONi010-C, KOLF2.1J and HMGU1 cell lines were used.
B. Volcano plot of differentially abundant proteins with increased abundance (purple) and decreased abundance (blue) proteins comparing VPA vs. Ctrl DFO samples at D35. The name of the selected proteins, SNCA, ANXA6 and ASTN1 can be found on the volcano plot.
C. Volcano plot of differentially abundant proteins with increased abundance (purple) and decreased abundance (blue) proteins comparing VPA vs. Ctrl DFO samples at D50.
D. Bar plot showing the number of differentially abundant proteins for VPA vs. Ctrl DFOs at D35 and D50.

For **B**, **C** and **D** the difference between time points is plotted against -log(P-value) with significance threshold: log2 Fold change value ≥1, FDR<0.05).

Figure S11

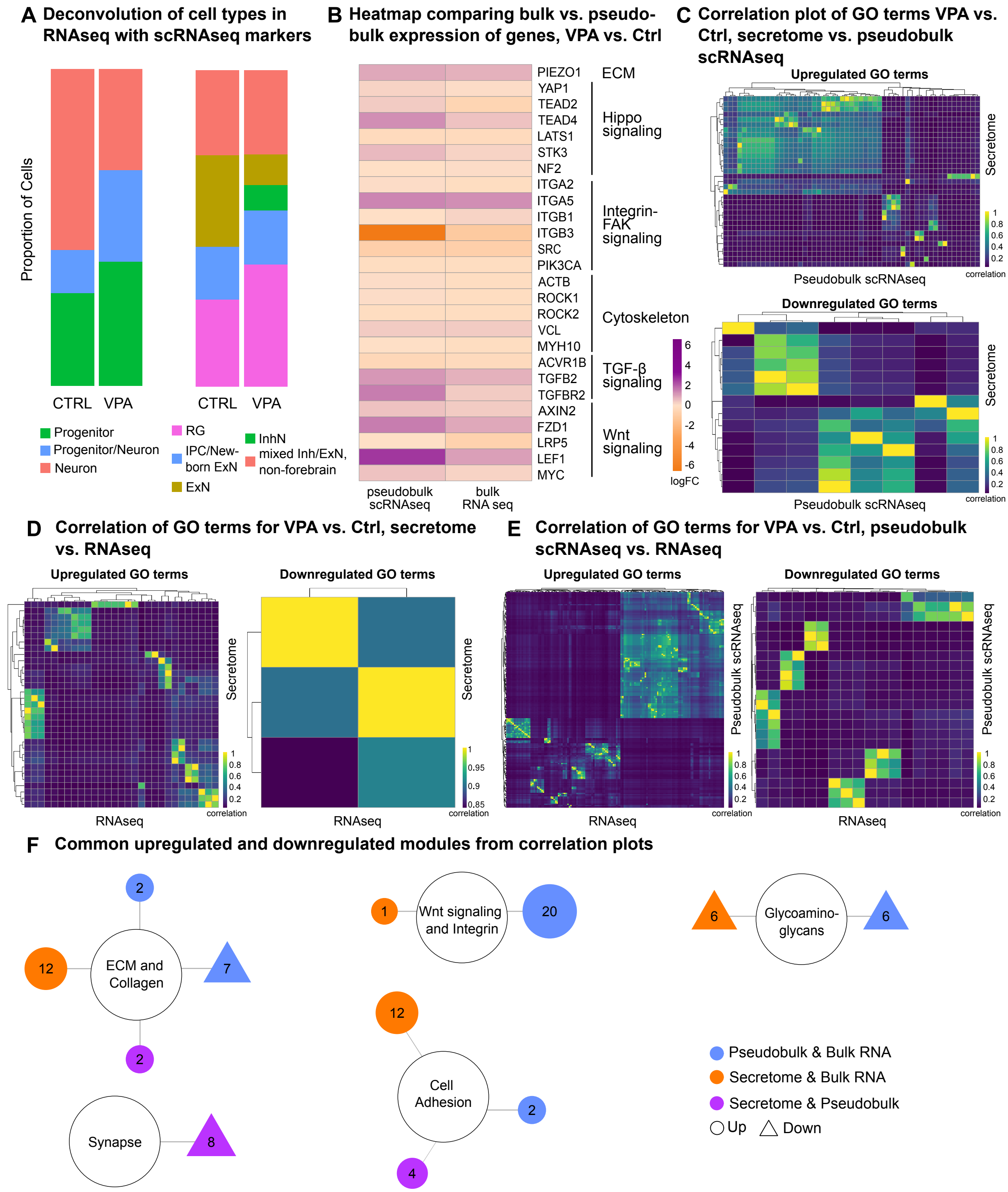

A Deconvolution of cell types in RNAseq with scRNAseq markers
Proportion of Cells
CTRL VPA
CTRL VPA
Progenitor
Progenitor/Neuron
Neuron
RG
IPC/New-born ExN
ExN
InhN
mixed Inh/ExN, non-forebrain
B Heatmap comparing bulk vs. pseudo-bulk expression of genes, VPA vs. Ctrl
PIEZO1
YAP1
TEAD2
TEAD4
LATS1
STK3
NF2
ITGA2
ITGA5
ITGB1
ITGB3
SRC
PIK3CA
ACTB
ROCK1
ROCK2
VCL
MYH10
ACVR1B
TGFB2
TGFBR2
AXIN2
FZD1
LRP5
LEF1
MYC
ECM
Hippo signaling
Integrin-FAK signaling
Cytoskeleton
TGF-β signaling
Wnt signaling
6
4
2
0
-2
-4
-6
logFC
pseudobulk scRNAseq
bulk RNA seq
C Correlation plot of GO terms VPA vs. Ctrl, secretome vs. pseudobulk scRNAseq
Upregulated GO terms
Secretome
Pseudobulk scRNAseq
correlation
Downregulated GO terms
Secretome
Pseudobulk scRNAseq
correlation
D Correlation of GO terms for VPA vs. Ctrl, secretome vs. RNAseq
Upregulated GO terms
Secretome
RNAseq
correlation
Downregulated GO terms
Secretome
RNAseq
correlation
E Correlation of GO terms for VPA vs. Ctrl, pseudobulk scRNAseq vs. RNAseq
Upregulated GO terms
Pseudobulk scRNAseq
RNAseq
correlation
Downregulated GO terms
Pseudobulk scRNAseq
RNAseq
correlation
F Common upregulated and downregulated modules from correlation plots
ECM and Collagen
2
12
7
2
Wnt signaling and Integrin
1
20
Glycoamino-glycans
6
6
Cell Adhesion
12
2
4
Synapse
8
Pseudobulk & Bulk RNA
Secretome & Bulk RNA
Secretome & Pseudobulk
Up
Down

Fig. S11: Integration of multiomics datasets comparing VPA-treated and Ctrl DFOs.
A. Deconvolution of cell types in bulk RNAseq dataset at D50 using the cell type markers found in the scRNA-seq dataset at D50.
B. Heatmap of gene expression of selected genes from relevant signaling pathways; ECM, Hippo signaling, integrin-FAK signaling and cytoskeleton using bulk RNAseq and scRNA-seq datasets. Log2FC has been plotted to indicate VPA vs. Ctrl comparison.
C. Heatmap for VPA vs. Ctrl correlating upregulated (top) and downregulated (bottom) GO terms from secretome (rows) and pseudobulk scRNA-seq (columns). Correlations of GO terms above 70% were plotted.
D. Heatmap for VPA vs. Ctrl correlating upregulated (left) and downregulated (right) GO terms from secretome (rows) and bulk scRNA-seq (columns). Correlations of GO terms above 70% were plotted.
E. Heatmap for VPA vs. Ctrl correlating upregulated (left) and downregulated (right) GO terms from pseudobulk scRNA-seq (rows) and bulk scRNA-seq (columns). Correlations of GO terms above 70% were plotted.
F. Diagram of common upregulated and downregulated modules from correlation plots. Selected recurrent modules were selected from the heatmaps **C-E** and the number of GO terms relevant were assessed. The size of the circle depends on the number of GO terms within the module; the color indicates the correlated datasets; the shape indicates whether the GO terms are upregulated or downregulated.

Figure S12

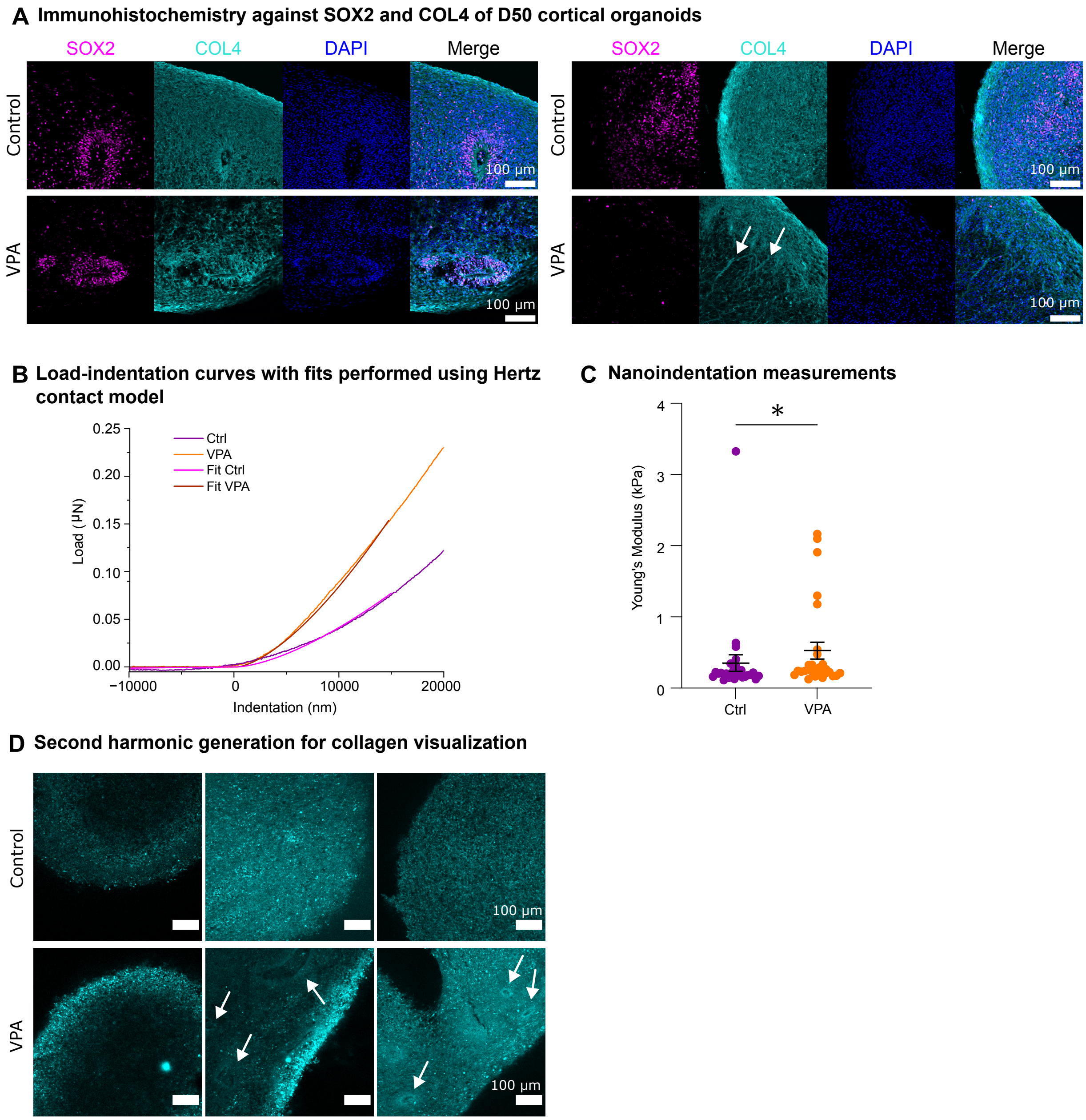
A Immunohistochemistry against SOX2 and COL4 of D50 cortical organoids
SOX2
COL4
DAPI
Merge
Control
VPA
100 µm
B Load-indentation curves with fits performed using Hertz contact model
Ctrl
VPA
Fit Ctrl
Fit VPA
Load (µN)
0.00
0.05
0.10
0.15
0.20
0.25
−10000
0
10000
20000
Indentation (nm)
C Nanoindentation measurements
Young's Modulus (kPa)
0
1
2
3
4
*
Ctrl
VPA
D Second harmonic generation for collagen visualization
Control
VPA
100 µm

Fig. S12: Extracellular matrix deposition in VPA-treated DFOs.
A. Representative image for SOX2 and COL4 immunohistochemistry of Ctrl and VPA-treated cortical organoids generated using a protocol from Pasca and Yoon (4) with modifications.
B. Two representative load-indentation curves with respective fits performed using Hertz contact model. The colors represent experimental conditions: purple, Ctrl; orange, VPA.
C. Individual recordings of Young's modulus (kPa) using nano-indentation method. The colors represent experimental conditions: purple, Ctrl; orange, VPA. Unpaired Mann-Whitney test was performed for statistical analysis using GraphPad software.
D. Imaging of Collagen 1 using second harmonic generation. Unique collagen depositions in VPA-treated organoids are marked with white arrows.
For **A-D**, one differentiation from BIONi010-C iPSC line was used.

Supplementary Tables

Table S1. Excel file containing information on experimental details and cell lines.

Table S2. Excel file containing VPA vs. Ctrl differentially expressed genes and GO terms from RNA sequencing data.

Table S3. Excel file containing VPA vs. Ctrl differentially expressed genes of each cell type from single cell RNA sequencing data.

Table S4. Excel file containing VPA vs. Ctrl GO terms derived from Table S3.

Table S5. Excel file containing VPA vs. Ctrl differentially abundant proteins from secretome and proteome of D35 and D50 VPA vs. Ctrl DFOs.

Table S6. Excel file containing VPA vs. Ctrl GO terms derived from Table S5.

References

1. Velasco S, Kedaigle AJ, Simmons SK, Nash A, Rocha M, Quadrato G, et al. Individual brain organoids reproducibly form cell diversity of the human cerebral cortex. Nature. 2019;570(7762):523-7.
2. Nano PR, Fazzari E, Azizad D, Nguyen CV, Wang S, Kan RL, et al. A Meta-Atlas of the Developing Human Cortex Identifies Modules Driving Cell Subtype Specification. bioRxiv. 2023.
3. Mato-Blanco X, Kim SK, Jourdon A, Ma S, Tebbenkamp ATN, Liu F, et al. Early Developmental Origins of Cortical Disorders Modeled in Human Neural Stem Cells. bioRxiv. 2024.
4. Pasca SPY, S. J. . Feeder-free, Xeno-free Generation of Cortical Spheroids From Human Pluripotent Stem Cells. Protocol exchange. 2018.
